# Emerging Applications of Nanotechnology in Dentistry

**DOI:** 10.3390/dj11110266

**Published:** 2023-11-15

**Authors:** Shiza Malik, Yasir Waheed

**Affiliations:** 1 Bridging Health Foundation, Rawalpindi 46000, Pakistan; shizzza874@gmail.com; 2 Office of Research Innovation and Commercialization (ORIC), Shaheed Zulfiqar Ali Bhutto Medical University (SZABMU), Islamabad 44000, Pakistan; 3 Gilbert and Rose-Marie Chagoury School of Medicine, Lebanese American University, Byblos 1401, Lebanon

**Keywords:** nanotechnology, nanodentistry, nanomaterials, nanocomposites, nanometals, dental nanomaterial and nanorobots, dentistry

## Abstract

Dentistry is a branch of healthcare where nanobiotechnology is reverberating in multiple ways to produce beneficial outcomes. The purpose of this review is to bring into the awareness of the readers the various practical dimensions of the nano-dental complex (nanodentistry) in healthcare and how novelties linked with the field are revolutionizing dentistry. A methodological approach was adopted to collect the latest data on nanotechnology and dentistry from sources, including PubMed, Google Scholar, Scopus, and official websites like the WHO. Nanodentistry is an emerging field in dentistry that involves the use of nanomaterials, nanorobots, and nanotechnology to diagnose, treat, and prevent dental diseases. The results summarize the descriptive analyses of the uses of nanodentistry within orthodontics, preventive dentistry, prosthodontics, restorative dentistry, periodontics, dental surgeries, dental restoration technologies, and other areas of dentistry. The future directions of nano-industries and nano-healthcare have been included to link them with the oral healthcare sector, treatment plans, and improved medical services which could be explored in the future for advanced healthcare regulation. The major limitations to the use of dental nanoproducts are their cost-effectiveness and accessibility, especially in financially constrained countries. These data will help the readers to experience a detailed analysis and comprehensive covering of the diverse achievements of nanodentistry with past analyses, present scenarios, and future implications.

## 1. Introduction

Healthcare biotechnology is a field that is always exploring more technological interventions for better healthcare management worldwide. Nanotechnology is one such essential part of healthcare that, along with other updated technologies, maintains an advanced outlook with practical implications in the field of medicine [1]. Nanodentistry is an emerging field in dentistry that involves the use of nanomaterials, nanorobots, and nanotechnology to diagnose, treat, and prevent dental diseases [2]. The focus of nanodentistry is to achieve precise and targeted delivery of therapeutic and diagnostic agents. Nanotechnology is a field of molecular manufacturing or engineering that involves structural adjustment of characteristics of materials, owing to the small size of nanomaterials within dimensions of 0.1–100 nm [3].

Dental health science offers opportunities to enhance patient satisfaction by improving the efficiency of dental tools, medications, and treatments. It also focuses on optimizing the kinetics reactions and mechanical strength of dental materials, resulting in stronger, more effective, and aesthetically pleasing products with better biocompatibility. Ultimately, this leads to easier and more efficient handling of dental products for the benefit of both practitioners and patients [3,4,5]. The vast scale applications of nanodentistry can be used in several dental procedures, such as cavity repair, teeth whitening, and orthodontic treatment [6,7]. It can also be used to improve the durability of dental restorations and to develop new materials that are stronger, more durable, and more biocompatible [8,9]. One of the most promising areas of nanodentistry is the development of nanorobots that can be programmed to perform certain dental procedures, such as cleaning and repairing teeth. These nanorobots can work at the molecular level and can even be controlled remotely using magnetic fields [10,11,12].

Overall, the focus of the present study is to explore how nanodentistry has revolutionized the field of dentistry, providing more precise and effective treatment options that are less invasive and more comfortable for patients. Additionally, the target of this study is to explore the implications of nanotechnology in dentistry within the aspects of diagnostics, preventive medicine, treatment options, restorative, and personalized medicines alongside cosmetics, industrial, and aesthetics applications [12,13]. This study will also highlight the improvement of dental materials with the passage of time that impart them with better mechanical properties, durability, antimicrobial activities, strengthening characteristics, pain-preventing techniques, and prompt recovery tactics [14,15]. We will also discuss some major implications of nano-dental materials, including nanocomposites, ceramics, glass ionomers, ceramics, metal needles, anesthetic components, and nanorobots, among others. In summary, a brief descriptive analysis will be presented to cover the tremendous applications of nanodentistry with the aspects of redefining and refreshing positive oral healthcare management. Overall, nanotechnology is a tool in the hands of researchers to explore a wide spectrum of continuous state-of-the-art nano-dental technology development with the hindsight aspects of better healthcare management.

## 2. Methodology

### 2.1. Search Criteria

The query method adopted was a search strategy on major search engines, like Google Scholar, PubMed, Scopus, and some official sites like the WHO, to collect relevant data mostly from the past 5 years, as we tried to limit data on the latest updates in the recent years. The keywords used for research were nanotechnology, nanodentistry, nanomaterials, nanocomposites, nanometals, dental nanomaterial, nanorobots, and dentistry, among others. As will be elaborated in the next section, around 100 relevant articles which focused on “Nanotechnology in Dentistry” were selected and explored for inclusion in the present study. Details on various search terms and the possible combination used for research on the above mentioned sites have been highlighted in Table 1.

### 2.2. Eligibility Criteria

The search items were limited to the keywords as described earlier, and the selected articles included peer reviews journals, reviews, research studies, short letters to the editors, and periodicals relevant to nanodentistry. Approximately 150–170 articles were initially accounted for during the search procedure; however, we tried to limit data derivation to the topmost cited and well-understood articles (around around 100 in total). Data lacking the details of articles, dissertations, theses, books, technical reports, or conference proceedings were avoided due to a lack of detailed accessibility and understanding. Only English language literature was made part of this review study for ease of global understanding.

## 3. Results

The results section will directly target discussion about a wide spectrum of applications of nanodentistry in dental technologies. But before coming to the application section, we first would like to briefly touch on the classification of nanomaterials based on physicochemical, shape, and composition-linked characteristics to elaborate upon which nanomaterials are most preferably used in nano-dental applications.

### 3.1. Classification of Nanoparticles in Dental Materials

Nanomaterials are differentiated by size and structural composition, which in turn dictates their physiochemical properties. Some of the major nanomaterials are briefly discussed below in table format. Table 2 discusses the general nanomaterials applied most in the dental industry [16,17,18,19,20]. Table 3 describes some nonconventional nanoparticles that are being explored for dental applications [21,22,23,24,25,26]. Table 4 shows the nanotube-based nanomaterials that are currently being explored for dental applications [9,26,27,28,29].

### 3.2. Application of Nanotechnology in Dentistry

#### 3.2.1. Operative Nanodentistry

##### Nano-Glass Ionomer

Classic glass ionomers combined with nanoparticles create nano-glass ionomers. Nonholonomic systems bring the characteristics of polishing, aesthetics, and improved wear resistance to the nanomaterials [2,59]. A bonded nanofiller technology-based formulation of ionomer cement is treated to enhance its mechanical properties by combination with other nanomaterials such as nanofillers, nanoclusters, and fluoroaluminosilicate glass [60]. These nanofillers improve the physical properties of teeth when used as hardened restorative material. They possess translucent properties, compressive strength, and better elasticity [13,58]. They have been shown to perform better than conventional glass ionomers. Additionally, their fluoride-releasing potential makes them suitable for application on surfaces with exposure to topical fluoride sources [18,58,63]. They are also effective in inhibiting caries upon acid exposure and can be used in various dental fillings [16]. Other applications of noncolumnar nanomaterials may include wide-ranging clinical indications, higher crystallization properties, sandwiched restorative technologies, and core buildups [26,67]. Bioactive substances, like hydroxyapatite and fluorapatite, are often used to produce better-class glass ionomers [55]. Some important examples include chitosan nanoparticles, nanohydroxyapatite, and nano-fluorapatite [55,56].

##### Nanocomposites

Nanocomposites are formed by combining two or more materials, including a matrix (polymer, metal, or ceramic) and nanoscale particles [68,69]. These non-agglomerated and discrete nanoparticle preparations can be homogenously distributed in dental resins and coatings to create composite materials for dental applications [69]. More effort has been put forth to improve the physical properties of composite materials that suffer from problems like polymerization shrinkage, weak coatings, triage risk, microhardness, and meager aesthetic satisfaction [66,69]. Nanocomposites are primarily used as dental materials with improved anti-decay potential. Dental surgeons prefer nanocomposites over conventional composite materials as they provide long-lasting tooth structural properties in patients [50,66,70]. The most widely used nanofillers in nanocomposites include aluminosilicate powder-based nanocomposites, which exhibit advanced physicochemical properties such as superior hardness, excellent flexure, best color density and polishing-retention ability, strength, improved modulus of elasticity, super translucency and good aesthetic appeal, and reduced filling shrinkage [31,40,48].

Dental nanocomposites are also used in restorative dentistry to mimic the properties of natural enamel. They add volume to composite resin and fillers, exhibit high strength and ion-releasing properties, prevent tooth decay, and restore fractures [71,72]. Moreover, they have a potential application as mineral provisions as they release fluoride phosphate and calcium when applied in increasing nano-positing formulations such as nano-dicalcium phosphate anhydrous (DCPA)-whiskers or tetra calcium phosphate (TTCP)-whiskers, and polymer-kaolinite nanocomposites [37].

The aesthetic application is pronounced for nanocomposites because of the reduced shrinkage and polymerization characteristics that increase filler volume and decrease resin matrix [1,73,74]. Moreover, they prevent tooth decay with increased fluorapatite and well-informed mechanical resistance. The nanocomposites also have high strength and ion-releasing properties which make them effective in preventing caries and restoring fractures [73,75]. The enrichment properties of nanocomposites directly facilitate remineralization and the mechanical strength benefits more from the additional amounts of nanofillers up to certain limitations [76]. There are some cases reported in which nanocomposite denture materials employ the use of inorganic fillers at the nanoscale, which are outstanding for their color retention, durability, and high abrasion resistance properties in comparison to the acrylic or microfiber compositions [77]. Examples of nanocomposites used in dentistry include Filtek Supreme, which contains glass particles and amorphous calcium phosphate (NACP) [6,77].

#### 3.2.2. Nanotechnology Applications in Prosthodontics

Prosthetic restorations, such as complete dentures made of polymethyl methacrylate resin or implants of acrylic particles, are commonly used for tooth recovery and oral rehabilitation [10]. Nanoparticles, such as Ag, ZrO_2_, and TiO_2_, are successfully employed in polymethyl methacrylate to treat problems like stomatitis caused by candida colonization [50,52,63]. Important examples of NPs application in prosthodontics include the combined application of TiO_2_ nanoparticles with three-dimensional poly-methyl methacrylate (PMMA) denture mixture, which enhanced antimicrobial properties and improved structural and chemical features [24,63].

Nano-zirconium oxide-modified heat-cured PMMA enhances the harness of denture, reduces aggregation, and improves biocompatibility, flexibility, transverse strength, and dispersion properties [50,52]. These nanomaterials are used in resin matrices and removable prosthodontics. Another application is the use of nanoparticles, such as sodium triphosphate (TP) or tri-metaphosphate (TMP), in chlorhexidine coatings for soft liner dentures. These coatings provide antimicrobial properties to fillings and coatings [63,64]. Some other nanomaterials used in prosthetics include nanoparticle-impregnated cement that improves bond strength to enamel and dentin. Zinc polycarboxylate with nanohydroxyapatite/fluorapatite particles exhibits higher tensile strength, biaxial flexure, and improved physical and mechanical properties compared to typical zinc polycarboxylate cement [63,64]. Furthermore, resin nanoceramic CAD/CAM blocks were recently launched and they showed improved tribological properties which allow for easily tailored impregnation in comparison to normal resin and ceramic [64,78]. The widespread application of these coatings in the future is promising due to their therapeutic necessity, extended lifespan, improved oral health, and cost-effectiveness [63]. However, comprehensive safety analyses and investigations into toxicity-related limitations should be carried out.

The use of nanomaterial-sized powders in prosthodontics is driven by their aesthetic value, superior wearing properties, and maximal strength compared to traditional ceramics [16]. Nano-glass ceramics, for instance, are strong, capable of withstanding bending loads, and are less abrasive to the opposing enamel. Adding AgNPs to dentures improves the treatment of stomatitis by preventing infection in the oral mucosa [34,35]. Other benefits of nanoparticles in prosthodontics include the use of nanofillers as polishing agents with reduced wearing, nano-pigments with restoration capabilities of surrounding teeth, and nanomodifiers with high stability and low adhesion to instruments [32,36,37].

#### 3.2.3. Nanotechnology Applications in Endodontics

Nanotechnology has brought remarkable advancements to endodontic dentistry. Nanoparticles are used in various endodontic applications, such as fillers, composites, irrigants, sealers, root repair materials, and photodynamic therapy, leading to beneficial outcomes [79]. Nanoparticles with antibacterial and anti-leakage properties are excellent for use as disinfectants in a wide range of dental applications, resulting in improved endodontic therapy [62]. Endodontic sealers contain different formulations of bio-ceramic nanoparticles like zirconia, glass ceramic, and bioglass, which provide strong bonding to dentin and release calcium and phosphate ions [23,50,52]. Examples of such sealers include Di-methylamino hexadecyl methacrylate (DMAHDM), amorphous calcium phosphate (ACP) nanoparticles, and 2-methacryloyloxyethyl phosphorylcholine (MPC) [2].

Similarly, adhesive nanoparticles also hold endodontic properties, offering fast settings, dimensional stability, reduction in nano-irregularities, and creating chemical connections and osteoconductivity in tooth restorations [62]. Moreover, NPs are helpful in remineralization by improving bonding strength and creating improved endodontic stresses [79]. Silver nanoparticles are often used to suppress bacterial growth in combination with calcium hydroxide intracanal medication, yielding quick and efficient results [34,35,36]. However, comprehensive profiling research is needed to determine the antibacterial properties of AgNPs against specific bacterial species [37]. Furthermore, the problem of managing bacterial biofilms that runs continuously in numerous dentinal tubules is extremely crucial in dental hygiene [56]. This is because these microflorae are resistant to conventional antiseptic measures due to their deep penetration into nanoscale root canals [56]. Metallic nanoparticles, therefore, provide an effective option for antiseptic application in root canals, overcoming persistent microflora. These sealers have better-dealing properties and act as efficient antimicrobial agents [35,80].

Other applications of nano-endodontics involve the incorporation of bio-ceramic nanoparticles like zirconia, glass, and bioglass NPs in endodontic sealers [50]. These NPs enable easy delivery, excellent sealing ability and biocompatibility, bioactivity, antimicrobial properties, and dimensional stability [41,80]. Ongoing research on state-of-the-art endodontic sealers includes Total Fill BC Sealer and Total Fill BC Sealer HiFlow, which have excellent bioactivity potential [79]. Additionally, GuttaFlow Bioseal is another silicon-based sealer containing a mixture of gutta-percha and calcium silicate particles along with some amount of finely grounded gutta-percha [79]. This product has been tested, exhibiting excellent adaptation and optimal flowability with gradual volumetric expansion, fine grain size, and a high thixotropic profile [2]. A nanodiamond particle incorporated into gutta-percha (GP) improves its traditional chemical, mechanical, and biocompatible properties. These nano-GPs are often used in high-quality endodontic filler to prevent reinfection, provide antibacterial action, and maintain dental health [16,17].

#### 3.2.4. Nanotechnology Applications in Orthodontics

The use of nanorobotics and nanocomposite formulations is advancing in the orthodontic industry. The use of nanorobots and nanoelectromechanical systems is useful in accelerating tooth repair and movement strategies, and it is supported by certain animal studies [27]. Similarly, studies have elaborated on the use of nanoparticle-based delivery systems (elastomeric ligatures) by realizing anti-cariogenic fluoride, bringing about anticancer use, antibiotics, and anti-inflammatory effects in the dental matrix [28]. Similarly, smart brackets with nanomechanical sensor technology proved to be more effective compared to conventional bracket devices, owing to the increased efficiency and timer perseverance of nanorobots [29]. Three-dimensional force movement systems have helped to increase the predictability of tooth movement and reduce traumatic side effects [81]. Orthodontic bracelets retain plaque and promote the formulation and proliferation of biofilms, reducing pH, and demineralizing the enamel; however, the addition of nanoparticles in these brackets inhibited plaque formation, reduced biofilms, increased the healing process, and induced an intimacy effect [74,81]. They also created a real-time feedback system to adjust the applied force in the biological range and reduce side effects [74].

#### 3.2.5. Nanotechnology Applications in Periodontics and Implantology

Regenerative nanodentistry, nano-periodontics, and nano-implantology are modern dental technologies that use nanomaterials, nanoscale structures, and techniques to repair and regenerate damaged or diseased teeth, gums, and bone tissues [22,31,82]. These fields of dentistry progressively employ nanoparticles in various combinational formulations. Regenerative nanodentistry involves the use of nanoscale materials and techniques to regenerate dentin, which is the inner layer of a tooth that forms the bulk of the tooth structure [59]. This technology involves the use of nanodiamonds, hydroxyapatite nanoparticles, and other nanomaterials that can stimulate the natural regenerative capacity of teeth to grow new layers of dentin and repair damaged or decayed teeth [55,56].

In this case, nano-periodontics technicians employ the use of nanomaterials and nanoscale structures to treat periodontal diseases, which affect the gums and the supporting bone tissues of the teeth [82]. This technology involves the use of nanoparticles that can penetrate deep into the gum tissues to kill bacteria, reduce inflammation, and promote tissue regeneration [59]. Similarly, nano-implantology uses nanoporous titanium surfaces to promote osseointegration, which is the process of fusing a dental implant with surrounding bone tissue [24]. This technology involves the use of nanoporous coatings that can increase the surface area of the dental implant, enhance the adhesion of bone cells to the implant surface, and promote faster healing and tissue regeneration [50,51].

More specifically, the use of tetracycline-loaded NPs or triclosan, such as noisome, possessing homogenous distribution profiles and extended periods of action, are used in drug delivery systems to treat periodontal tissues [53,83]. Additionally, the use of fullerenes (hollow carbon molecules) as antioxidants and radical scavengers in general medical and specific dental fields is being explored for similar drug delivery purposes [53,83]. Moreover, bone grafting with the help of light-curable methacrylate resin matrix and nACP, which crystallize back into hydroxyapatite in a few minutes, is also under scientific research [65]. Gold, silver titanium oxide, and hydroxyapatite nanoparticles help to increase implant integration, improve matrix surface topography, and create nano-groves and nanopillars [34,55,56]. For regenerative purposes, root canal therapy is assisted by nanoparticle conjugation to remove necrosis pulp tissues and replace these with healthy pulp tissue. Testing on melanocortin peptides (MSH) with anti-inflammatory properties showed positive effect on pupal proliferation [16,22].

Additionally, dental coatings in the implant industry often contain nanoparticles due to their unique reformative properties and nano-topography [1,60]. These NPs, along with other implant materials, play a critical role in exhibiting biocompatibility and bio-integrative properties with dentin tissues [19,31]. NPs help to modify an implant’s surface characteristic, like surface roughness, topography, surface energy, and composition, which influence bone–implant interfaces [52]. With successful trials of NP-based dental implants and the defined electrical, magnetic, optical, and mechanical properties of NPs, implant technologies are being further developed to create varying surface textures to influence tissue responses [24]. These NPs also exhibit structural characteristics such as grain boundaries, interphase boundaries, and dislocation within the material, which dictate varying chemical and physical properties [51,62].

Moreover, ongoing research in tissue engineering concepts and periodontal regeneration technologies utilizes synthetic scaffolds of NPs for cell delivery purposes [22]. There is a hope to develop nonbiologic self-assembling systems for tissue engineering that can create nanodomains and phases with built-in nano-control and delivery capabilities. If research continues in the field of nano-tissue engineering, titanium-based dental implants, which have been in use for the past three decades, can overcome their previous limitations in osseointegration properties. The latest inclusion of nano-sized titanium oxide formulations was in improving the osteointegration and physiological properties of titanium-based implants [24,50,51,75]. Similarly, antimicrobial peptides, such as LL37, can be immobilized along with medical apparatuses in dental implants to provide the best antimicrobial and antigenic properties [51].

Periodontitis is a bacterial infection that cause inflammation, resulting in tooth loosening and loss. Previously, the available treatment options were limited to mechanical removal of pathogenic biofilms and drug-based local and systematic treatments [59]. However, the current trends based on micro- and nanoparticles are changing the game for quick and efficient treatment [82]. Nano-based drugs have a longer shelf life, better bioactivity, controlled release of microspheres, antibiotic effect, and sealing and healing properties. Overall, these advanced dental technologies have the potential to revolutionize dental care by offering more effective, efficient, and minimally invasive treatments for a wide range of dental conditions [7,59].

#### 3.2.6. Nanotechnology Applications for Hypersensitivity Management

Hypersensitivity is a common tooth issue that occurs due to tooth root exposure. One approach to treat hypersensitivity is the use of dentine tubules that seal and isolate tooth roots from external pain stimuli [1,84,85]. The inclusion of nanorobots in dentine tubules increasingly occludes the specific sensitive tubules in patients, permanently curing the hypersensitivity issue [86]. Gold NPs are most widely used in such dental tubules. Moreover, reconstructive dental nanorobots can also selectively regulate hypersensitivity by blocking microtubules, desensitization, and reducing the volume of microtubules in mineralizing agents [13,33,34].

#### 3.2.7. Nanotechnology Applications for Nano-Tissue Engineering

Nanoparticles represent the forefront of tissue engineering in the medical field. Bone tissue engineering and nanotechnology have revolutionized deontology by providing innovative solutions to a range of dental problems [40]. Bone tissue engineering involves the use of biodegradable scaffold materials that mimic the structure and function of natural bone. These scaffolds are combined with stem cells and growth factors to promote the growth of new bone tissue [40,83]. In dentistry, both technologies have been used to develop new materials for dental implants, fillings, and other restorative procedures [31]. The biocompatible nature of these materials has also greatly improved the success rates of these procedures. For example, researchers have developed a nanostructured material that can promote the growth of new bone tissue in the jaw, improving the success of dental implants [19,52].

Nanoparticles have also been used to deliver antibiotics directly to bacterial infections in the gums, improving treatment outcomes and reducing the risk of resistance [35]. Though some obstacles remain in regenerative techniques, like impaired cellular proliferation and differentiation, and insufficient manufacturing of extrinsic elements which plays a vital role in osteogenesis, these cannot deny the use of bone tissue engineering and nanotechnology that has led to significant improvements in dental treatment outcomes by providing innovative solutions to a range of dental problems [41,50]. As these technologies continue to advance, we can expect even more exciting developments in the field of deontology.

#### 3.2.8. Nanotechnology Applications in the Surgical Field

Nanotechnology has shown promising potential in the surgical field. More specific examples may include nanorobots for surgery (nanobots) which are tiny robots that can move through tissues and organs [80]. They can be programmed to perform various surgery-related tasks, including drug delivery, tissue biopsies, and blood clot removal [53]. Similarly, NPs can be utilized for advanced wound healing where nano-engineered materials can promote faster wound healing and tissue regeneration [16,87]. For example, silver nanoparticles are known for their antimicrobial properties that can prevent infections. Nanoparticles coated with stem cells have also been shown to promote tissue regeneration [22]. Similarly, nanoparticles can be used as drug carriers to deliver drugs directly to an affected area. This reduces the amount of medication needed and minimizes negative side effects [53,88].

Moreover, in imaging and diagnostics, nanoparticles can be used to improve diagnostic accuracy. For instance, gold nanoparticles are used to enhance contrast in imaging techniques, such as CT and MRI scans [34,48]. Additionally, smart prosthetics and implants, already explained in the earlier section, is a field where nanotechnology can facilitate the development of smart prosthetics and implants that can respond to natural biochemical signals and adjust their function accordingly [10,63]. This can improve patients’ mobility and reduce the risk of complications. Overall, nanotechnology has the potential to revolutionize the surgical field by improving treatment outcomes, reducing complications, and enhancing patient comfort [64].

Furthermore, in the case of nano-anesthetic applications, colloidal solutions of millions of nano-sized active analgesic nanobots are installed in patients’ gingiva along with chemical applications for dental surgeries and other procedures [53]. These dental nanorobots are then controlled by surgeons in terms of the content and quality of reactions at particular times and sites of reaction [53,83]. After procurement, normal dental functionalities are restored. Moreover, nanotechnologies are used in bone deformational corrections, wherein nanomaterials with their unique properties also show promising results for the treatment of bone and teeth deformities [5,65]. The major nanophase material used for this purpose includes nanophase hydroxyapatite and nanophase carbon, which show excellent properties of osteoblastic adhesion, maxillofacial-implanting capabilities, and general biomedical properties as compared to conventional materials [55,56].

#### 3.2.9. Preventive Nanodentistry

Preventive nanodentistry is a branch of dentistry that utilizes nanotechnology to prevent tooth decay and other oral health problems. It involves the use of nano-sized particles and materials to prevent or repair damage to teeth at the molecular level [87]. Some of the applications of preventive nanodentistry include nanocomposite fillings which are made of nanometer-sized particles that mimic the natural mineral structure of teeth [37]. They are stronger, more durable, and less likely to fracture than traditional composite fillings. Similarly, nanoparticle coatings are applied to teeth to prevent bacteria from sticking to them, thus reducing the risk of tooth decay and gum disease [4,87]. The application of nanoparticles in toothpaste is also a novel method by which toothpaste is manufactured to contain nanometer-sized particles that can penetrate the tooth enamel and provide a protective barrier against cavities [85]. Similarly, tiny nano-sensors can detect early signs of oral health problems, such as tooth decay or gum disease, allowing for early intervention and treatment. Along with this, nanogels can be applied to the teeth to re-mineralize or repair damaged enamel, thus preventing cavities and tooth decay [85].

Preventive nanodentistry has the potential to revolutionize the field of dentistry by providing more effective and efficient ways to prevent and treat oral health problems. It could improve overall oral health outcomes and help to reduce the need for invasive procedures [17,87]. Moreover, the use of modern nonidentity preventers, rather than treating biofilm formation, is a major problem in different oral diseases like caries, endodontics, and periodontics diseases [87]. The use of therapeutic, prophylactic toothpaste and nano-toothbrushes and mouthwashes containing NP formulations is also being promoted to encourage remineralization and prevent tooth decay, plaque, caries, and odor impact at the nanoscale level [4,37]. Examples of these dental hygiene products may include mouth wash containing nano-calcium fluoride and nano-hydroxyapatite (NHA), as well as toothpaste containing calcium carbonate nanoparticle [80]. These products not only act superficially but also penetrate deep into the natural surfaces to have positive impacts on teeth and gums [17].

#### 3.2.10. Nanotechnology Applications in Diagnosis

In dentistry, nanotechnology has the potential to revolutionize diagnosis and treatment by allowing for more accurate and efficient diagnostic techniques. Nanotechnologies are being used in dental diagnosis, including in the use of smart dental probes as tiny, nanoscale sensors that can detect early signs of tooth decay by analyzing the chemical composition of a tooth’s surface [14]. Smart probes can generate images of a tooth’s surface, providing dentists with more accurate and detailed information than conventional X-rays [14,53,83]. Nanobiosensors, as tiny nanoscale devices, can detect biomolecules in saliva that are associated with various oral diseases. Biosensors can quickly identify bacterial or fungal infections, making it easier for dentists to diagnose and treat conditions [52]. Similarly, the vast field of nanoparticle imaging enables nanoparticles to highlight areas of inflammation or disease in the mouth [89]. Nanoparticles can also be used to target cancer cells, making it easier for dentists to identify and treat oral cancer [90,91]. Similarly, nano-based drug delivery systems can be used to deliver drugs directly to diseased or damaged areas of the mouth, reducing the need for invasive procedures. This approach can help minimize side effects and enhance the effectiveness of treatments [87].

In these various ways, nanotechnology enables advanced research methods in the field of dental science. More recently, dental laser technology has introduced a new optical phenomenon, which when irrigated in the oral cavity easily penetrates to micropores at the foci of demineralization [14,65]. Such methods have proven effective in the detection and pathogenesis of diseases. Other diagnosis devices may include biosensor technologies carrying biological diagnostic elements with wide-scale implementation potential in oncology [4,51]. Overall, nanotechnologies are opening exciting possibilities for more precise and efficient diagnosis and treatment in dentistry. As research advances, we can expect to see more innovative uses for this technology in dental care [25].

#### 3.2.11. Nano-Molecular Imaging in Dental Science

Nano-molecular imaging in dentistry refers to the use of advanced imaging technologies to visualize the molecular and cellular changes that occur in oral tissues and lesions [36]. This type of imaging can provide dentists with invaluable information about the nature and progression of dental diseases, such as caries, periodontitis, and oral cancer [32,91]. One of the most promising nano-molecular imaging techniques being developed for dentistry is called nano-optical coherence tomography (OCT) [65]. This technology uses near-infrared light to create high-resolution, three-dimensional images of dental tissue at the nanoscale level, enabling dentists to see structures and cellular changes that occur in teeth and gums [92]. Other imaging techniques that are being explored for dentistry include confocal microscopy, atomic force microscopy, computed tomography (CT), magnetic resonance imaging (MRI), OCT, and fluorescent spectroscopy [93]. These approaches can provide dentists with detailed information about molecular and biochemical markers of oral diseases, as well as the ability to track the progression of these diseases over time [54,93].

In techniques such as MRI and CT scan, NPs increase spatial resolution, improve the contrast of tissues, infinitely enhance penetration depth, and improve metabolic imagining. There are limitations, like selective agent sensitivity, inadequate contrasting, and sensitivity toward specific agents [11,94]. Additionally, photoacoustic (PA) imaging serves as a non-invasive technique in the optical diagnostics field, where an advanced level of optical imaging is used for clinical research and medical producers [95]. These methods also serve as biomarkers for oral cancer detection with the help of tumor marker biomarkers [14,96]. However, there is a need to investigate the cytotoxic effects of higher concentrations when used for cancer studies. Overall, nano-molecular imaging is an exciting area of research in dentistry, with the potential to revolutionize the diagnosis and treatment of oral diseases [12]. As these technologies continue to develop, we can expect to see more accurate diagnoses, more effective treatments, and improved oral health outcomes for patients [12,82,97].

#### 3.2.12. Some Other Applications of Nanodentistry

Dental adhesives are a subcategory of restorative dentistry that are used for invasive dentistry. Nano-adhesives, nano-sized fillers, and nano-reinforced bonding agents allow for technicians to design more conservative designs for cavity protection, improve mechanical properties, and promote healthy tissue by acting as adhesives between composting materials and resins [12,49,98]. An important example of nano-adhesives includes Adper™ Single Bond Plus Adhesive (St. Paul, MN, USA). Similarly, the class of surface disinfection has been revolutionized by nanotechnology, which incorporates the use of nano-emulsions to develop nano-sized droplets against pathogens for surface disinfection [32,99]. These nanoparticles kill small microorganisms, including viruses, and can be used in sterilization and incision technologies to prevent postoperative infections [15,100].

Another important application is nano-stainless steel needles and crystals, which are used mostly for manufacturing surgical sutures, enabling efficient dental and periodontal surgeries at the microscale level [8,13]. Other newly introduced dimensions may include nano-piers, nano-forceps, and nanoneedles, which provide surface detailing and improved molding properties for surgical equipment [15]. Finally, nano-dental ultrafine teeth polishing helps to reduce tooth roughness and medium biofilm formation. It also protects against pathogenic bacteria and carcinogenic microorganisms, while promoting superior-level aesthetics and dental-restoration effects [98,100].

### 3.3. Limitations in the Field

Although nanotechnology has tremendous applications in the field of dentistry, it is important to acknowledge the limitations of its continuous utilization. These limitations are biological, chemical, mechanical, social, and ethical, among others as shown in Table 5 [1,16,17]. The first challenge is the cost management and accessibility of nano-dental products. An additional limitation is marketing these products in countries where economic as well as research and development sectors are slow, and the healthcare sectors already suffer from financial constraints [18]. Another challenge is developing biocompatible nanomaterials to ensure compatibility with the human body. Due to their smaller size, nanoparticles can easily pass the blood–brain barrier, human skin cells, and lungs, posing potential health concerns [19,20]. Moreover, the oral surface may become hypersensitive and hyperactive towards nanoparticle emulsions, leading to hypersensitive reactions [21,65].

Enzymatic profiles may also be altered in some cases, as demonstrated by previous research experiments. Moreover, upon further cellular integration, nanoparticles can cause free radicalization, cellular toxicity, enhanced pro-inflammatory responses, and oxidative stress [21,22,23]. Proper regulation of the systemic reactions of nano-based drugs and treatments is necessary to avoid adverse reactions in the body. Extensive research in animal models is needed due to the highly active and sometimes toxic nature of nanomaterials, especially metal nanoparticles, to ensure human safety [23]. Moreover, there may be social xenophobia towards new and unknown technologies, which hinders the realization of a nanotechnology-driven future in the medical field unless appropriate measures are taken [61]. Ethical concerns are linked to issues such as patient approval, family consent, dosage consideration, prior animal testing, and informed consent for human experimentation. Therefore, overcoming these prevailing challenges is crucial for adopting a balanced perspective on a nanotechnology-based future of dental sciences.

## 4. Future Perspectives and Conclusions

This article has demonstrated how nano-dental science has contributed to improving the quality, appearance, durability, wearing properties, resistance, sensitivity, and hyperactivity of teeth. It is expected that nanotechnology will continue to grow and find more applications in the field of health science. This prediction is based on the potential benefits and cost-effectiveness; the socio-environmental, public, and occupational advantages; and the risks associated with nano-dental science. Over time, researchers have been increasingly exploring new dimensions of nano-dental science while working to overcome its limitations. The aim of future research should be on overcoming the limitations attached to the nano-dental industry, and adaptation of the sociocultural and economic agenda within the periphery of nanodentistry clinical applications. It is anticipated that nanodentistry will replace conventional treatment methods in the near future. During this period, it is crucial for scientists to remain dedicated to nano-research, address limitations, conduct safety analyses, and develop marketing, acceptability, and promotional strategies for practical approval.

## Figures and Tables

**Table 1:** Search items and combinations used for driving data for review.

Sr. No.	Search Items	Search Combinations
1.	Nanotechnology	Dentistry and nanotechnology Applications of nanotechnology in dentistry Update on nanodentistry
2.	Nanodentistry	Nanodentistry benefits and application Latest update on nanoscience application in dental field
3.	Nanomaterials	Nanomaterials used in dental sciences Nanomaterials and dentistry
4.	Nanocomposites	Applications of nanocomposites in dentistry
5.	Nanometals	Nanometals and dentistry
6.	Nanoscineeces and healthcare	Healthcare and nanomaterials; latest applications Applications of nanomaterials in healthcare and dentistry
7.	Nanorobots	Nanorobots applications in field of dentistry

**Table 2:** Classification of nanoparticles in dental materials.

Sr. No.	Nanomaterials	Properties	Benefits and Applications	References
1.	Nanoparticles	Conventional or unconventional.	Easily manufactured; Flexible in application.	[30,31,32]
2.	Metallic nanoparticles	Antibacterial characteristics.	Infections treatment.	[33,34]
3.	Silver nanoparticles (AgNPs)	Antibacterial, antiviral, and antifungal properties; Induce microorganisms cell death; Prevent secondary caries; Easy combination with; bioactive substances; Strengthen dental composites; Flexural strength and elastic modulus; Improve dental health; Biocompatible; Long-lasting antibacterial activity.	Restorative materials; Dental fillers; Dental aesthetics; Endodontic sealers; Remineralization; Dental restorative materials; Dental implants, dental prostheses.	[34,35,36,37]
4.	Carbon nanotubes	Increased surface area; Active substance delivery to cells; Quick attachment to the tooth and cementum surface.	Tooth fillers; Tooth coatings.	[38,39,40]
5.	Graphene-based NPs	Cost-effective; Fracture-resistant; Low-density; Homogeneous crystal lattice structure; Effective against bacterial biofilms.	Tooth coatings; Tooth implantations; Biofilm reduction.	[41,42]
6.	Gold nanoparticles (AuNPs)	Inert; Biocompatible; Antibacterial properties; Stimulate bone growth; Preserve bone formation; Chemical functionalization.	Nano-drug delivery systems; Treating and detecting cancers; Photothermal agents; Contrast agents; Nano-drug delivery carriers; Osteo inductive agents; Dental implants.	[33,34,36]
7.	Copper nanoparticles (CuNPs)	Antimicrobial activities; Prevent adhesive interface; Maintain formulation properties.	Dental materials.	[43]
8.	Metal oxide nanoparticles	More stable as compared to metal NPs; Antibacterial properties.	Dental fillings.	[33,44,45,46]
9.	Zinc oxide nanoparticles (ZnO NPs)	Antibacterial efficacy; Less cytotoxic effects.	Dental composite resins; Dental materials.	[47,48,49]
10.	Titanium dioxide nanoparticles (TiO_2_ NPs)	High strength; Corrosion resistance; Excellent biocompatibility; Antibacterial properties.	Titanium alloys; Composite resins.	[24,50,51]
11.	Titania-based NPs	Long-term effect on dental implants; Decreased bacterial adherence; Increased hardness.	Dental implants.	[24,50,51]
12.	Zirconium dioxide nanoparticles (ZrO_2_ NPs)	Rigidity; Improved radio-capacity; Fatigue resistance; Wear resistance; Biocompatibility; Osteo conductivity; Decreased plaque buildup; Strength; Bending resistance; Good appearance; Improved fracture toughness; Color stability.	Cosmetic applications; Fillers.	[50,52]
13.	Aluminum oxide nanoparticles (Al_2_O_3_ NPs)	Sensitive to sunlight, heat, and moisture; Polished surface wear resistance; Hardness, good biocompatibility; Improved mechanical strength; Improved mechanical and thermal properties; Reduced water absorption and solubility; Reduced friction.	Aesthetics applications.	[1,50,53]
14.	Silicon dioxide nanoparticles (SiO_2_ NPs)	Improved mechanical qualities; Low toxicity; Good biocompatibility.	Fillers; Dental restorative materials; Polishing agents; Medicinal compounds.	[1,16,54]
15.	Zirconia	Reduced bacterial adhesion; Protection against caries; Excellent polisher; Comparable mechanical properties and color; Low cytotoxicity; Good biocompatibility and great fracture resistance.	Dental polishers; Natural.	[50,52]
16.	Silica-based NPs	Prevent dental caries; Biocompatible; Minimal toxic effect; Low density; High adsorption capacity; Cost-effective; Reduce tooth roughness.	Dental filling agents’ dental hypersensitivity treatment; Antibacterial agents and tooth polishing.	[35,54]
17.	Hydroxyapatite (HAp)-based NP formulations.	Easier incorporation in tubules; Comparative tooth and bone properties; Biocompatible; Enamel adhesion; Tooth protection; Reduced hypersensitivity; Delay secondary demineralization.	Cavity fillers; Tooth repairing creams.	[55,56,57]

**Table 3:** Nanoparticles used in the field of dentistry.

Sr. No.	Unconventional Nanoparticles	Characteristics	Applications	References
1.	Nanodiamonds	Excellent surface properties; Compatible chemical nature.	Dental nanocomposite; Fillers; Root canal treatments; Dental prostheses; Restorative dentistry; Polymer reinforcement; Tissue regeneration; Drug administration; Dental implant coatings.	[20,58]
2.	Quantum dots	Semiconductive properties; Light emission; Conjugation properties.	Nanocarriers; Drug delivery; Genetic treatments; Anticancer medicine; Diagnostic imaging; Prevents oral cancer.	[2,55,58,59]
3.	Nanoshells	Anti-cancerous properties; Targeted medication delivery.	Therapeutic purposes; Reducing angiogenesis; Promoting wound healing; Limiting internal bleeding.	[13,55,60]
	Quaternary ammonium methacrylate (QAM) nanoparticles	Antibacterial properties; Increase osmotic pressure.	Restorative dental material; 3D biofilms; Dental adhesive.	[17,18,61]
	Quaternary ammonium polyethyleneimine (QPEI) nanoparticles	Prevent root canal infections; Antibacterial properties.	Antibacterial sealers; Endodontic sealers; Resin composites.	[62,63]
	Amorphous calcium phosphate nanoparticles (ACP NPs)	Antimicrobial effects; Tooth natural properties retention; Remineralization; Maintain pH levels.	Dentin and enamel remineralization; Restoring materials.	[5,10,64]
	Nanoplatelet-based nanomaterials	Made from nanoplatelets and nanosheets.	Ideal for various dental applications.	[14,20,65,66]

**Table 4:** Important nanotube-based nanomaterials.

Sr. No.	Nanotube-Based Nanomaterials	Properties	Applications	References
1.	Carbon nanotubes (CNTs)	Exceptional mechanical and electrical properties; Flexural strength; Anticancerous properties; High impact strength.	Dental composites; Fillers; Dental implants; Anticancer medication; Drug delivery vehicle.	[38,40]
2.	Halloysite nanotubes (HNTs)	Natural milky white color; Antibacterial properties; Elastic modulus; High strength; Prevent secondary dental caries.	Dental fillers; Nano-drug delivery agents; Dental composting; Antibacterial drugs.	[2,15,59]
3.	Graphene oxide nanoplatelets	Improved mechanical properties; High antibacterial properties; Least cytotoxic effect; Increased bone production; Decreased inflammatory reactions; Excellent biocompatibility.	Graphene nano-powder; Dental nanocomposites; Fillers; Antibacterial agents; Tissue engineering; Regenerative dentistry; Graphene oxide (GO)-coated It (GO-Ti) membranes; Dental coatings and implants.	[41,42]

**Table 5:** Limitations attached to nanotechnological applications in dentistry.

Biological Limitations	Chemical and Mechanical Limitations	Social Limitations	Ethical Limitations	Economic Limitations
Developing bio-friendly nanomaterials; Managing healthcare concerns; Extensive research needed.	Ensuring nanotechnological biocompatibility; Toxicity profiling and safety analyses.	Accessibility and acceptability; Overcoming social xenophobia.	Patients approval; Family consent; Dosage; Consideration and prior animal testing; Informed consent for human experimentation.	Marketing issues; Cost management; Financial constraints; Customer retention programs.

## References

[1] Jandt K.D., Watts D.C. (2020). Nanotechnology in Dentistry: Present and Future Perspectives on Dental Nanomaterials. Dent. Mater..

[2] Malik S., Muhammad K., Waheed Y. (2023). Emerging Applications of Nanotechnology in Healthcare and Medicine. Molecules.

[3] Barot T., Rawtani D., Kulkarni P. (2021). Nanotechnology-Based Materials as Emerging Trends for Dental Applications. Rev. Adv. Mater. Sci..

[4] Amissah F., Andey T., Ahlschwede K.M. (2021). Nanotechnology-Based Therapies for the Prevention and Treatment of Streptococcus Mutans-Derived Dental Caries. J. Oral Biosci..

[5] Gauba K., Gupta A., Sharda S. (2022). Nanotechnology in Dentistry. Biomed. Transl. Res. Drug Des. Discov..

[6] Subramani K., Elhissi A., Subbiah U., Ahmed W. (2019). Introduction to nanotechnology. Nanobiomaterials in Clinical Dentistry.

[7] Kennedy S., Srinivasan S., Jayavel K., Sundaram R. (2019). Nanotechnology in Periodontal Management. Int. J. Orofac. Biol..

[8] Contera S., Bernardino de la Serna J., Tetley T.D. (2020). Biotechnology, Nanotechnology and Medicine. Emerg. Top. Life Sci..

[9] Deepali S., Priyanka A., Shweta B., Kirti P. (2021). Nanotechnology in periodontics. A review. Santosh Univ. J. Health Sci..

[10] Patel R.M., Dahane T.M., Godbole S., Kambala S.S., Mangal K. (2020). Applications of Nanotechnology in Prosthodontics. J. Evol. Med. Dent. Sci..

[11] Soto F., Wang J., Ahmed R., Demirci U. (2020). Medical Micro/Nanorobots in Precision Medicine. Adv. Sci..

[12] Buniyamin I., Akhir R.M., Asli N.A., Khusaimi Z., Malek M.F., Mahmood M.R. (2022). Nanotechnology Applications in Biomedical Systems. Curr. Nanomater..

[13] Mirsasaani S.S., Hemati M., Dehkord E.S., Yazdi G.T., Poshtiri D.A. (2019). Nanotechnology and nanobiomaterials in dentistry. Nanobiomaterials in Clinical Dentistry.

[14] Joseph B. (2023). Nanotechnology in Oral and Dental Diagnosis. Nanomaterials in Dental Medicine.

[15] Haleem A., Javaid M., Singh R.P., Rab S., Suman R. (2023). Applications of Nanotechnology in Medical Field: A Brief Review. Glob. Health J..

[16] Foong L.K., Foroughi M.M., Mirhosseini A.F., Safaei M., Jahani S., Mostafavi M., Ebrahimpoor N., Sharifi M., Varma R.S., Khatami M. (2020). Applications of Nano-Materials in Diverse Dentistry Regimes. RSC Adv..

[17] Prabakar J. (2023). Current applications of nanoparticles in preventive dentistry–A Literature review. J. Surv. Fish Sci..

[18] Kochan O., Boitsaniuk S., Levkiv M., Przystupa K., Manashchuk N., Pohoretska K., Chornij N., Tsvyntarna I., Patskan L. (2022). Emergence of Nano-Dentistry as a Reality of Contemporary Dentistry. Appl. Sci..

[19] Agnihotri R., Gaur S., Albin S. (2019). Nanometals in Dentistry: Applications and Toxicological Implications—A Systematic Review. Biol. Trace Elem. Res..

[20] Uppal M.K., Sharma M.L., Sharma S., Thakar S. (2021). The Magnanimity of Nanodentistry.

[21] Sreenivasalu P.K.P., Dora C.P., Swami R., Jasthi V.C., Shiroorkar P.N., Nagaraja S., Asdaq S.M.B., Anwer M.K. (2022). Nanomaterials in Dentistry: Current Applications and Future Scope. Nanomaterials.

[22] Dissanayaka W.L., Sharpe P. (2023). Editorial: Frontiers in Dental Medicine: Highlights in Regenerative Dentistry 2021/22. Front. Dent. Med..

[23] Calisir M. (2019). Nanotechnology in dentistry: Past, present, and future. Nanomaterials for Regenerative Medicine.

[24] Guo T., Scimeca J.-C., Ivanovski S., Verron E., Gulati K. (2023). Enhanced Corrosion Resistance and Local Therapy from Nano-Engineered Titanium Dental Implants. Pharmaceutics.

[25] Nandagopal N., Usha M., Sreejith S., Rajan S. (2021). A Clinical Review of Nanotechnology in Maxillofacial Practice. J. Oral Res. Rev..

[26] Umapathy V.R., Natarajan P.M., SumathiJones C., Swamikannu B., Johnson W.M.S., Alagarsamy V., Milon A.R. (2022). Current Trends and Future Perspectives on Dental Nanomaterials—An Overview of Nanotechnology Strategies in Dentistry. J. King Saud Univ. Sci..

[27] Padmanabhan S. (2023). Nanotechnology in Orthodontics. Semin. Orthod..

[28] Ali Fathima S., Abiraj K.R., Pratheesha A.P., Mohan S.M., Krishna A.S., Dynamol S. (2023). Knowing the unknown: A review on nanotechnology in Orthodontics. J. Pharm. Res. Int..

[29] Zakrzewski W., Dobrzynski M., Dobrzynski W., Zawadzka-Knefel A., Janecki M., Kurek K., Lubojanski A., Szymonowicz M., Rybak Z., Wiglusz R.J. (2021). Nanomaterials application in Othrodontics. Nanomaterials.

[30] Bhushan J., Maini C. (2019). Nanoparticles: A Promising Novel Adjunct for Dentistry. Indian J. Dent. Sci..

[31] Hossain N., Islam M.A., Chowdhury M.A., Alam A. (2022). Advances of Nanoparticles Employment in Dental Implant Applications. Appl. Surf. Sci. Adv..

[32] Vasiliu S., Racovita S., Gugoasa I.A., Lungan M.-A., Popa M., Desbrieres J. (2021). The Benefits of Smart Nanoparticles in Dental Applications. Int. J. Mol. Sci..

[33] Chandra H., Kumari P., Bontempi E., Yadav S. (2020). Medicinal Plants: Treasure Trove for Green Synthesis of Metallic Nanoparticles and Their Biomedical Applications Biocatal. Agric. Biotechnol..

[34] Sakthi Devi R., Girigoswami A., Siddharth M., Girigoswami K. (2022). Applications of Gold and Silver Nanoparticles in Theranostics. Appl. Biochem. Biotechnol..

[35] Wang Q., Zhang Y., Li Q., Chen L., Liu H., Ding M., Dong H., Mou Y. (2022). Therapeutic Applications of Antimicrobial Silver-Based Biomaterials in Dentistry. Int. J. Nanomed..

[36] Fernandez C.C., Sokolonski A.R., Fonseca M.S., Stanisic D., Araújo D.B., Azevedo V., Portela R.D., Tasic L. (2021). Applications of Silver Nanoparticles in Dentistry: Advances and Technological Innovation. Int. J. Mol. Sci..

[37] Butrón Téllez Girón C., Hernández Sierra J.F., DeAlba-Montero I., Urbano Peña M.d.l.A., Ruiz F. (2020). Therapeutic Use of Silver Nanoparticles in the Prevention and Arrest of Dental Caries. Bioinorg. Chem. Appl..

[38] Castro-Rojas M.A., Vega-Cantu Y.I., Cordell G.A., Rodriguez-Garcia A. (2021). Dental Applications of Carbon Nanotubes. Molecules.

[39] Mohamed P.A., Fahmy A.E., El Shabrawy S.M. (2023). Three-Dimensionally Printed Denture Base Resins Modified by Nanoglass Particles and Carbon Nanotubes. J. Prosthet. Dent..

[40] Mousavi S.M., Yousefi K., Hashemi S.A., Afsa M., BahranI S., Gholami A., Ghahramani Y., Alizadeh A., Chiang W.-H. (2021). Renewable Carbon Nanomaterials: Novel Resources for Dental Tissue Engineering. Nanomaterials.

[41] Radhi A., Mohamad D., Abdul Rahman F.S., Abdullah A.M., Hasan H. (2021). Mechanism and Factors Influence of Graphene-Based Nanomaterials Antimicrobial Activities and Application in Dentistry. J. Mater. Res. Technol..

[42] Nizami M.Z.I., Takashiba S., Nishina Y. (2020). Graphene Oxide: A New Direction in Dentistry. Appl. Mater. Today..

[43] Xu V.W., Nizami M.Z.I., Yin I.X., Yu O.Y., Lung C.Y.K., Chu C.H. (2022). Application of Copper Nanoparticles in Dentistry. Nanomaterials.

[44] He L., Dai D., Xie L., Chen Y., Zhang C. (2021). Biological Effects, Applications and Strategies of Nanomodification of Dental Metal Surfaces. Mater. Des..

[45] Yazdanian M., Rostamzadeh P., Rahbar M., Alam M., Abbasi K., Tahmasebi E., Tebyaniyan H., Ranjbar R., Seifalian A., Yazdanian A. (2022). The Potential Application of Green-Synthesized Metal Nanoparticles in Dentistry: A Comprehensive Review. Bioinorg. Chem. Appl..

[46] Thangavelu L., Veeraragavan G.R., Mallineni S.K., Devaraj E., Parameswari R.P., Syed N.H., Dua K., Chellappan D.K., Balusamy S.R., Bhawal U.K. (2022). Role of Nanoparticles in Environmental Remediation: An Insight into Heavy Metal Pollution from Dentistry. Bioinorg. Chem. Appl..

[47] Pushpalatha C., Suresh J., Gayathri V., Sowmya S., Augustine D., Alamoudi A., Zidane B., Mohammad Albar N.H., Patil S. (2022). Zinc Oxide Nanoparticles: A Review on Its Applications in Dentistry Front. Bioeng. Biotechnol..

[48] Moradpoor H., Safaei M., Mozaffari H.R., Sharifi R., Imani M.M., Golshah A., Bashardoust N. (2021). An Overview of Recent Progress in Dental Applications of Zinc Oxide Nanoparticles. RSC Adv..

[49] Raja V., Selvan G., Anbarasu R., Baskar S. (2019). Synthesis of Zinc Oxide Nanoparticles and Setariaverticillata Assisted Activated Carbon Blended Zinc Oxide Nanoparticles. Indian J. Sci. Technol..

[50] Karthikeyan M., Ahila S.C., Kumar B.M. (2019). The antibacterial influence of nanotopographic titanium, zirconium, and aluminum nanoparticles against Staphylococcus aureus and porphyromonas gingivalis: An in vitro study. Indian J. Dent. Res..

[51] Gulati K., Ding C., Guo T., Guo H., Yu H., Liu Y. (2023). Craniofacial Therapy: Advanced Local Therapies from Nano-Engineered Titanium Implants to Treat Craniofacial Conditions. Int. J. Oral Sci..

[52] Gupta S., Noumbissi S., Kunrath M.F. (2020). Nano Modified Zirconia Dental Implants: Advances and the Frontiers for Rapid Osseointegration. Med. Devices Sens..

[53] Nagarale R., Kadu N., Dhumavat P., Muluk S.S., Jamal A. (2022). Nano Anesthesia and Nano Drug Delivery-A Review Article. Asian J. Dent. Sci..

[54] Ha S.-W., Weiss D., Weitzmann M.N., Beck B.R. (2019). Applications of silica-based nanomaterials in dental and skeletal biology. Nanobiomaterials in Clinical Dentistry.

[55] Bordea I.R., Candrea S., Alexescu G.T., Bran S., Băciuț M., Băciuț G., Lucaciu O., Dinu C.M., Todea D.A. (2020). Nano-Hydroxyapatite Use in Dentistry: A Systematic Review. Drug Metab. Rev..

[56] Sinjari B., Pizzicannella J., D’Aurora M., Zappacosta R., Gatta V., Fontana A., Trubiani O., Diomede F. (2019). Curcumin/Liposome Nanotechnology as Delivery Platform for Anti-Inflammatory Activities via NFkB/ERK/pERK Pathway in Human Dental Pulp Treated With 2-HydroxyEthyl MethAcrylate (HEMA). Front. Physiol..

[57] Altwaijri Y.A., Al-Habeeb A., Al-Subaie A.S., Bilal L., Al-Desouki M., Shahab M.K., Hyder S., Sampson N.A., King A.J., Kessler R.C. (2020). Twelve-month Prevalence and Severity of Mental Disorders in the Saudi National Mental Health Survey. Int. J. Methods Psychiatr. Res..

[58] Vellaichamy M., Škarabot M., Muševič I. (2023). Optical Gain and Photo-Bleaching of Organic Dyes, Quantum Dots, Perovskite Nanoplatelets and Nanodiamonds. Liq. Cryst..

[59] Samanta S. (2022). Periodontics: ORIGINAL REVIEW Nanoperiodontics-A Futuristic Trend in Periodontal Management. Clin. Dent. (0974-3979).

[60] Malik S., Muhammad K., Waheed Y. (2023). Nanotechnology: A Revolution in Modern Industry. Molecules.

[61] Chokkattu J.J., Neeharika S., Rameshkrishnan M. (2023). Applications of Nanomaterials in Dentistry: A review. J Int Soc Prev Community Dent..

[62] Raura N., Garg A., Arora A., Roma M. (2020). Nanoparticle Technology and Its Implications in Endodontics: A Review. Biomater. Res..

[63] Gupta R., Sharma S. (2022). Nanotechnology in Prosthetic Dentistry A Review. J. Prosthet. Implant Dent..

[64] Choubisa D. (2022). An Overview of Applications of Nanotechnology in Prosthodontics. J. Prosthodont. Dent..

[65] Sen S., Singh G. (2020). Finding Hidden Gems: Nanoparticles in Oral Health-A Review. Int. J. Drug Res. Dent. Sci..

[66] Sen D., Patil V., Smriti K., Varchas P., Ratnakar R., Shetty D.K., Naik N., Kumar S. (2022). Nanotechnology and Nanomaterials in Dentistry: Present and Future Perspectives in Clinical Applications. Eng. Sci..

[67] Altankhishig B., Matsuda Y., Nagano-takebe F., Okuyama K., Yamamoto H., Sakurai M., Naito K., Hayashi M., Sano H., Sidhu S.K. (2022). Potential of Fluoride containing Zinc Oxide and Copper Oxide nanocomposites on dental bonding ability. Nanomaterials.

[68] Gupta P.K. (2023). Applications of Nanotechnology in Dentistry. Nanotoxicol. Nanobiomed..

[69] Asha A.B., Narain R. (2020). Nanomaterials properties. Polymers Science and Nanotechnology. Fundam. Appl..

[70] Hamouda R.A., Qarabai F.A.K., Shahabuddin F.S., Al-Shaikh T.M., Makharita R.R. (2023). Antibacterial activity of Ulva/Nanocellulose and Ulva/Ag/Cellulose nanocomposites and both blended with Fluoride against bacteria causing dental decay. Polymers.

[71] Bonilla-Represa V., Abalos-Labruzzi C., Herrera-Martinez M., Guerrero-Pérez M.O. (2020). Nanomaterials in Dentistry: State of the Art and Future Challenges. Nanomaterials.

[72] Orts-Gil G., Natte K., Österle W. (2013). Multi-Parametric Reference Nanomaterials for Toxicology: State of the Art, Future Challenges and Potential Candidates. RSC Adv..

[73] Xenaki V., Marthinussen M.C., Costea D.E., Didilescu A.C., Susin C., Cimpan M.R., Åstrøm A.N. (2019). Knowledge about Nanotechnology and Intention to Use Nanomaterials: A Comparative Study among Dental Students in Norway and Romania. Eur. J. Dent. Educ..

[74] Tahmasbi S., Mohamadian F., Hosseini S., Eftekhar L. (2019). A review on the applications of nanotechnology in orthodontics. Nanomed J..

[75] Zafar M.S., Amin F., Fareed M.A., Ghabbani H., Riaz S., Khurshid Z., Kumar N. (2020). Biomimetic Aspects of Restorative Dentistry Biomaterials. Biomimetics.

[76] Modi S., Prajapati R., Inwati G.K., Deepa N., Tirth V., Yadav V.K., Yadav K.K., Islam S., Gupta P., Kim D.-H. (2021). Recent Trends in Fascinating Applications of Nanotechnology in Allied Health Sciences. Crystals.

[77] Zafar M.S., Alnazzawi A.A., Alrahabi M., Fareed M.A., Najeeb S., Khurshid Z. (2019). Nanotechnology and nanomaterials in dentistry. Advanced Dental Biomaterials.

[78] Park E.Y., Kang S. (2020). Current Aspects and Prospects of Glass Ionomer Cements for Clinical Dentistry. Yeungnam Univ. J. Med..

[79] Zakrzewski W., Dobrzyński M., Zawadzka-Knefel A., Lubojański A., Dobrzyński W., Janecki M., Kurek K., Szymonowicz M., Wiglusz R.J., Rybak Z. (2021). Nanomaterials Application in Endodontics. Materials.

[80] Esteban Florez F.L., Trofimov A.A., Ievlev A., Qian S., Rondinone A.J., Khajotia S.S. (2020). Advanced Characterization of Surface-Modified Nanoparticles and Nanofilled Antibacterial Dental Adhesive Resins. Sci. Rep..

[81] De Stefani A., Bruno G., Preo G., Gracco A. (2020). Application of Nanotechnology in Orthodontic Materials: A State-of-the-Art Review. Dent. J..

[82] Qi M., Li X., Sun X., Li C., Tay F.R., Weir M.D., Dong B., Zhou Y., Wang L., Xu H.H.K. (2019). Novel Nanotechnology and Near-Infrared Photodynamic Therapy to Kill Periodontitis-Related Biofilm Pathogens and Protect the Periodontium. Dent. Mater..

[83] Makvandi P., Josic U., Delfi M., Pinelli F., Jahed V., Kaya E., Ashrafizadeh M., Zarepour A., Rossi F., Zarrabi A. (2021). Drug Delivery (Nano) Platforms for Oral and Dental Applications: Tissue Regeneration, Infection Control, and Cancer Management. Adv. Sci..

[84] Panchbhai A. (2019). Nanotechnology in dentistry. Applications of Nanocomposite Materials in Dentistry.

[85] Moothedath M., Moothedath M., Jairaj A., Harshitha B., Baba S., Khateeb S. (2019). Role of Nanotechnology in Dentistry: Systematic Review. J. Int. Soc. Prev. Community Dent..

[86] Sachdeva S., Mani A., Mani S.A., Vora H.R., Gholap S.S., Sodhi J.K. (2021). Nano-robotics: The future of health and dental care. IP Int. J Perio Implant..

[87] Kumar R., Jha K., Barman D. (2021). Nanotechnology in Oral Cancer Prevention and Therapeutics: A Literature Review. Indian J. Med. Paediatr. Oncol..

[88] Tawfik E.A., Tawfik A.F., Alajmi A.M., Badr M.Y., Al-jedai A., Almozain N.H., Bukhary H.A., Halwani A.A., Al Awadh S.A., Alshamsan A. (2022). Localizing Pharmaceuticals Manufacturing and Its Impact on Drug Security in Saudi Arabia. Saudi Pharm. J..

[89] Mani R., Chellapandian K., Gayathri K., Kumar S.K., Kondas V.V. (2022). Role of Nanotechnology in Regeneration of Pulpo-Dentinal Complex. Int. J. Health Sci..

[90] Lubojanski A., Dobrzynski M., Nowak N., Rewak-Soroczynska J., Sztyler K., Zakrzewski W., Dobrzynski W., Szymonowicz M., Rybak Z., Wiglusz K. (2021). Application of Selected Nanomaterials and Ozone in Modern Clinical Dentistry. Nanomaterials.

[91] Babu N.A., Anjuga E.P.S., Nagarajan K., Masthan K.M.K. (2019). Nanotechnology in Detection of Oral Cancer. Indian J. Public Health Res. Dev..

[92] Tetè G., Capparè P., Gherlone E. (2020). New Application of Osteogenic Differentiation from HiPS Stem Cells for Evaluating the Osteogenic Potential of Nanomaterials in Dentistry. Int. J. Environ. Res. Public Health.

[93] Jandt K.D., Sigusch B.W. (2009). Future Perspectives of Resin-Based Dental Materials. Dent. Mater..

[94] Liu F., Hong T., Xie J., Zhan X., Wang Y. (2021). Application of Reactive Oxygen Species-Based Nanomaterials in Dentistry: A Review. Crystals.

[95] Yazdanian M., Rahmani A., Tahmasebi E., Tebyanian H., Yazdanian A., Mosaddad S.A. (2021). Current and Advanced Nanomaterials in Dentistry as Regeneration Agents: An Update. Mini-Rev. Med. Chem..

[96] Mok Z.H., Proctor G., Thanou M. (2020). Emerging Nanomaterials for Dental Treatments. Emerg. Top. Life Sci.

[97] Kumara Sundaram R., Varghese B. (2020). All Ceramic Materials in Dentistry: Past, Present and Future: A Review. Int. J. Contemp. Med. Res. [IJCMR].

[98] Kalotra J., Gaurav K., Kaur J., Sethi D., Arora G., Khurana D. (2020). Recent Advancements in Restorative Dentistry: An Overview. J. Curr. Med. Res. Opin..

[99] Vijayalakshmi R., Ramakrishnan T., Srinivasan S., Kumari B.N. (2020). Nanotechnology in Periodontics: An Overview. Medico-Legal Update.

[100] Bastos N.A., Bitencourt S.B., Martins E.A., De Souza G.M. (2020). Review of Nano-technology Applications in Resin-based Restorative Materials. J. Esthet. Restor. Dent..

